# Pain and psyche in a patient with irritable bowel syndrome: chicken or egg? A time series case report

**DOI:** 10.1186/s12876-021-01879-2

**Published:** 2021-08-03

**Authors:** Felicitas Engel, Tatjana Stadnitski, Esther Stroe-Kunold, Sabrina Berens, Rainer Schaefert, Beate Wild

**Affiliations:** 1 Department of General Internal Medicine and Psychosomatics, University of Heidelberg, Im Neuenheimer Feld 410, 69120 Heidelberg, Germany; 2 Department of Quantitative Methods in Psychology, University of Ulm, Albert-Einstein-Allee 47, 89081 Ulm, Germany; 3 Department of Psychosomatic Medicine, Division of Internal Medicine, University Hospital Basel, Hebelstrasse 2, 4031 Basel, Switzerland

**Keywords:** Functional gastrointestinal disorders, Irritable bowel syndrome, Time series analysis, Case report, Single case study

## Abstract

**Background:**

Irritable bowel syndrome (IBS) appears to have a bidirectional interaction with both depressive and anxiety-related complaints. However, it remains unclear how exactly the psychological complaints, at the individual level, are related to somatic symptoms on a daily basis. This single case study investigates how somatic and psychological variables are temporally related in a patient with irritable bowel syndrome.

**Case report:**

The patient was a woman in her mid-twenties with an IBS diagnosis. She reported frequent soft bowel movements (5–6 times per day), as well as flatulence and abdominal pain. She resembled a typical IBS patient; however, a marked feature of the patient was her high motivation for psychosomatic treatment as well as her willingness to try new strategies regarding the management of her symptoms. As an innovative approach this single case study used a longitudinal, observational, time series design. The patient answered questions regarding somatic and psychological variables daily over a period of twelve weeks with an online diary. The diary data was analysed using an autoregressive (VAR) modeling approach. Time series analyses showed that in most variables, strong same-day correlations between somatic (abdominal pain, daily impairment) and psychological time series (including coping strategies) were present. The day-lagged relationships indicated that higher values in abdominal pain on one day were predictive of higher values in the psychological variables on the following day (e.g. nervousness, tension, catastrophizing, hopelessness). The use of positive thinking as a coping strategy was helpful in reducing the pain on the following days.

**Conclusion:**

In the presented case we found a high correlation between variables, with somatic symptoms temporally preceding psychological variables. In addition, for this patient, the use of positive thoughts as a coping strategy was helpful in reducing pain.

## Background

Irritable bowel syndrome (IBS) is characterized by recurrent abdominal pain that is associated with a change in frequency or form (appearance) of stool and can be related to defecation [1]. Currently, the symptom pattern is not sufficiently explained by peripheral organ pathology. IBS affects about 8% of the European population [2] and is most recently understood as a disorder of (microbiota-) gut-brain interaction [3, 4] with a multifactorial origin that includes biological, psychological, and social factors [5]. Many patients who suffer from IBS also suffer from comorbid depressive or anxiety-related disorders [5]. Mood and anxiety disorders can precede or follow an IBS diagnosis due to the high discomfort caused by IBS [6–8]. By looking at specific psychological variables it was found that catastrophizing is directly associated with IBS symptom severity, while anxiety is indirectly related to IBS symptom severity [9].

While population-based studies suggest that IBS has a bidirectional interaction with both depressive and anxiety-related complaints, it remains unclear how exactly the psychological complaints, at the individual level, are related to somatic symptoms on a daily basis. Are increased psychological complaints (such as depression, tension, and nervousness) on one day preceded by IBS complaints on the previous day, or is it the other way around? A previous study showed that week-to-week stress and IBS symptoms were strongly cross-correlated in the same week, but were not temporally related across several weeks [10]. However, a day-by-day measure is needed to identify more fine-grained and direct relations. Furthermore, the focus of the study was on the mean values from a large patient sample, therefore potentially differing relationships in individual patients may not have been reflected in the aggregated data analysis.

Another interesting topic in patients suffering from IBS is the mutual relationship between coping strategies and IBS symptoms. A recent study reported that levels of coping resources were associated with gastrointestinal and extraintestinal symptom severity [11]. Also, catastrophizing and a lower self-perceived ability to reduce symptoms appeared to have a negative effect on health outcome in gastrointestinal disorders [12]. Interestingly, IBS patients have been reported to use passive coping strategies more frequently (such as escape-avoidance strategies instead of intended problem solving) compared to healthy controls [13]. Here too, the question arises to what extent coping strategies are related to IBS complaints and whether or not they are able to influence IBS complaints.

Overall, IBS symptoms and psychological distress are bi-directionally related, and coping strategies purportedly play an important role in the up- or down-regulation of IBS symptoms. However, individual mechanisms are not yet understood, and previous studies lack the longitudinal data on a day-by-day basis. Longitudinal data is necessary in order to obtain information about direct interactions, to better understand how temporal interactions between IBS symptoms and psychological complaints are related. As aggregated data can eliminate individual effects within the heterogeneous IBS patient sample, a single case study can provide important insights into specific mechanism to generate hypotheses for personalized clinical studies [14]. Conversely, inferences from singe case studies do not automatically apply to the patient population. However, results from single case studies can be used to generate hypotheses that can be examined in a sample of patients with similar characteristics.

This case study has, for the first time, applied a longitudinal time series design to a patient with IBS. Study objectives of this single-case analysis were: (1) to explore temporal relationships and interactions between the somatic and psychological complaints of the patient and (2) to investigate the impact of personal coping strategies on somatic symptoms.

## Case presentation

### Study design

The study used a longitudinal, observational, single-case design. The study was approved by the medical ethics committee of the University Hospital Heidelberg. The patient was recruited in the frame of a pilot intervention study, conducted between July, 2014 and June, 2015 [15]. During her waiting period—and before the beginning of the therapy group—the patient answered questions daily regarding somatic and psychological complaints as well as coping strategies with the use of an online diary.

The diary data of the patient was collected following presentation in our outpatient specialty clinic for functional gastrointestinal disorders [16], and before group therapy. The data thereby showed the classic course of IBS without specific group intervention. The patient filled out the diaries from 10/2014 to 01/2015; over twelve weeks a total of 72 diary days were collected.

### Measurements in the online diary

At the beginning of the study the patient received individual training in how to use the online diary; she was instructed to fill out the diary on a daily basis (between 4 pm and 12 am) via internet access. Validated questionnaires for IBS, as well as for psychological complaints and coping strategies, were used and adapted for the daily diary design. The most discriminating items of the questionnaires were derived in order to shorten the completion time of the diary (approximately 5–10 min). All items were rated by a visual analogue scale (VAS) with bipolar labels. The marked points were then converted by the computer program to a numeric scale ranging from 1 to 101. In addition, it was possible to enter a short free text in the diary.

For the measurement of somatic symptoms, we used the items “How severe is your abdominal (tummy) pain?” and “Please indicate how much your irritable bowel syndrome is affecting or interfering with your life today?”. Higher scores on these items reflected higher pain or higher somatic impairment. Psychological variables and coping strategies measured in the online diary are shown in Table 1.

**Table 1:** List of Online-Diary Items included in the time series analysis

	Items implemented in the online diary
**Somatic variables**	
Abdominal pain (AP)	“How severe is your abdominal (tummy) pain” → Adapted from the irritable bowel severity scoring system (IBS-SSS) [17]
IBS associated daily impairment (DI)	“Please indicate how much your irritable bowel syndrome is affecting or interfering with your life today” → Adapted from the irritable bowel severity scoring system (IBS-SSS) [17]
**Psychological variables**	
Nervousness (N)	“Today, how much were you distressed by nervousness or shakiness inside?” → Adapted from the brief symptom inventory (BSI) [18]
Tension (T)	“Today, how much were you distressed by feeling tense or keyed up” → Adapted from the brief symptom inventory (BSI) [18]
Depressiveness (D)	“Today, how often have you been bothered by feeling down, depressed, or hopeless?” → Adapted from the Patient-Health-Questionnaire (PHQ) [19]
Pain associated discomfort (PD)	“Today, how much have you been bothered by stomach pain” → Adapted from the Patient-Health-Questionnaire (PHQ) [20]
**Coping strategies**	
Catastrophizing (C)	“Today, when experiencing IBS-pain you had the feeling that you couldn’t go on” → Adapted from the coping strategies questionnaire (CSQ) [21]
Hopelessness (H)	“When you had IBS-pain today, you thought: “It’s terrible and I feel it’s never going to get any better” → Adapted from the coping strategies questionnaire (CSQ) [21]
Coping: positive thoughts (CPT)	“Today, when experiencing IBS-pain I thought of things I enjoy doing” → Adapted from the coping strategies questionnaire (CSQ) [21]
Coping: Imagining pain outside the body (CIP)	“When experiencing IBS-pain, today I imagined that the pain is outside of my body” → Adapted from the coping strategies questionnaire (CSQ) [21]

All the variables are quantified on a 1 to 101 numeric scale. For AP, DI, N, T, D, PD, C, and H a higher score reflects higher somatic or psychological burden. For CPT and CIP, a higher score reflects an increased use of coping strategies

### Case report

In January 2014, a German student (female in her mid-twenties), was referred to the outpatient specialty clinic of the University Hospital of Heidelberg for functional gastrointestinal disorders. She reported frequent soft bowel movements (5–6 times per day), as well as flatulence and abdominal pain. According to ROM-III [22] and the clinical assessment, an IBS (subtype IBS-diarrhea, IBS-D) was diagnosed. In addition, the patient was suffering from comorbid gluten, lactose, and sorbitol intolerance. No mental illness was present. Despite professional nutritional advice that included a gluten-, lactose- and sorbitol-reduced diet, gastrointestinal complaints persisted. In the course of the three-month follow-up appointments that included multimodal treatment [16] (04/2014, 07/2014, 11/2014), the patient correlated intestinal complaints and stress. She reported, for example, that the intestinal symptoms increased at the beginning of the semester and in the examination period. In the course of the diary study the patient did not describe any long-lasting stressor (such as an examination phase), but rather shorter week- or day-specific stressful events (such as Christmas holidays or looking for a part-time job) associated with an onset of IBS-symptoms on the same day. As an additional stressor, she described shame and the fear of a recurrence of the IBS complaints (particularly of soft bowel movements and flatulence), especially in social settings and situations where she could not easily reach a toilet. Relaxation techniques (yoga and gut-directed hypnosis using a CD) slightly improved her symptoms and the associated fear. Regarding the short stressful events, she described a good improvement of symptoms when using a strategy of calming down, with no further subsequent exacerbation. After the online diary study presented here, the patient received a group intervention [15] from which she has benefited.

In conclusion, according to IBS symptoms, symptom specific fears and avoidance behavior, the presented case of a young female patient resembled a typical IBS patient; however, a marked feature of the patient was her high motivation for psychosomatic treatment as well as her willingness to try new strategies regarding the management of her symptoms.

### Statistical analysis

Initially, the following analyses were conducted for each time series: graphic examinations; calculations of descriptive statistics (range, median, mean, standard deviation), autocorrelation functions (ACF), and tests for stationarity with the Augmented Dickey–Fuller (ADF) procedure. Autocorrelation is the bivariate correlation of a time series with a lagged copy of itself. Therefore, instantaneous (lag = 0) autocorrelation is always equals one, significant autocorrelations on other lags imply predictability of the future time series values from the past values. Stability or instability as well as memory characteristics of time series can be inferred from their autocorrelation functions: non-zero autocorrelations at only a few lags are typical for stable short-memory processes, whereas significant autocorrelations on many lags indicate long memory or instability. Stationarity means that the statistical characteristics of a process under study do not change over time (e.g., exhibit no trends or distinct fluctuations of mean or variance). The Augmented Dickey-Fuller algorithms tests the null hypothesis “time series is stationary”.

In addition, cross-correlation functions (CCF), instantaneous correlations, and simultaneous regressions with psychological measures—both as dependent and somatic variables as predictors—were estimated. Cross-correlation measures similarity of two different time series as a function of the displacement of one relative to the other. Generally, instantaneous (lag = 0) correlations or simultaneous (lag = 0) regressions do not imply causation. For lagged correlations and regressions, however, it is different, since they explore the ability to predict the future values of a time series from prior values of another times series. The idea behind this is as follows: Since time does not run backwards, the cause cannot come after its effect. Therefore, events in the past can cause events to happen today, but future events cannot influence the present. The concept of Granger causality incorporates this idea: if lagged values of a time series X improve prediction of future values of a series Y, the former series Granger-causes the latter. For example, if lagged values of a somatic times series improve prediction of future values of a psychological one, the former series Granger-causes the latter. The vector autoregressive (VAR) methodology investigated the temporal dynamics between two or more time series by separating the time-lagged from the simultaneous relations. Therefore, temporal interdependencies between time series were analyzed using this approach. The VAR technique thereby allowed inferences about the temporal order of the effects by employing the temporal causality concept introduced by Granger. Furthermore, the VAR approach can handle time series that mutually influence each other and thus reveal feedback effects. In VAR modelling, interpretation of the regression coefficients is problematic because the lagged values of the dependent variables are used as predictors (i.e. dependent and independent variables are both endogenous, that is, determined and interrelated inside the organism or system), consequently, external influences can enter the autoregressive system exclusively through the residual term, which is also called “exogenous shock”. The behaviour of a VAR system can be modelled using impulse response analyses (IRA) and forecast error variance decompositions (FEVD). Impulse response functions (IRF) examine interdependencies within a VAR system by tracing the effect of an exogenous shock in one of the series on other variables. The FEVD estimates the amount of variance in each variable that can be explained by the other variables of the system during a specific period (h). For instance, in case of daily measurements, FEVD = 0.24 (h = 10) means that 24% of the forecast error variance in a dependent variable can be explained by exogenous shocks (random changes) of the predictors for a time horizon of 10 days.

The analyses were conducted using the R software. (Please consult Stadnitski & Wild (2019) and Stadnitski (2014, 2020) for descriptions, detailed explanations, and implementation of all analyses with the R software [23–25]).

### Results

**Fig. 1:**
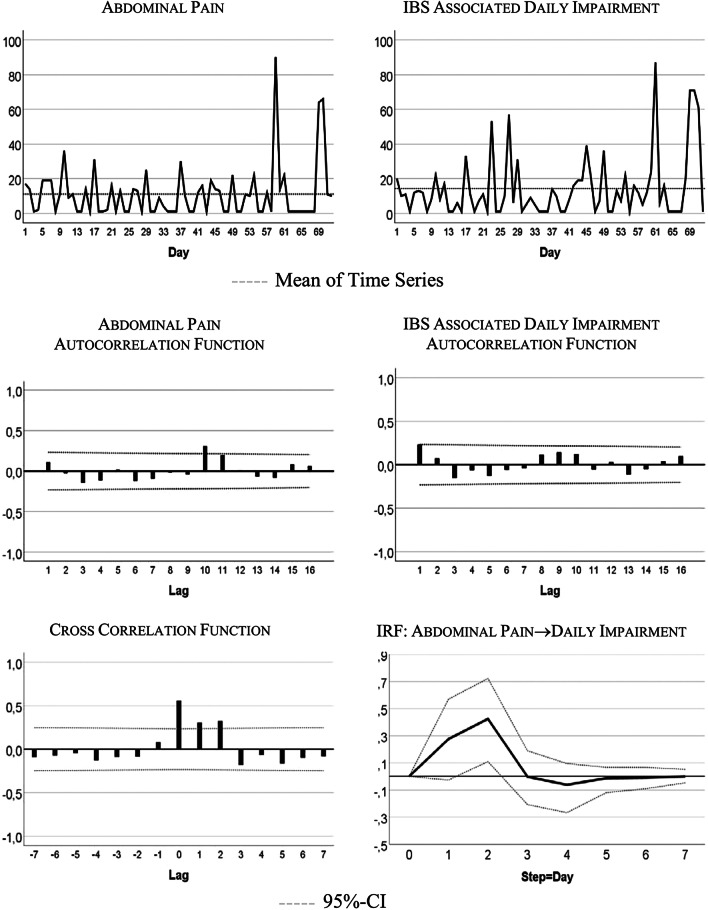
Somatic time series: abdominal pain (AP) and IBS-associated daily impairment (DI)

**Fig. 2:**
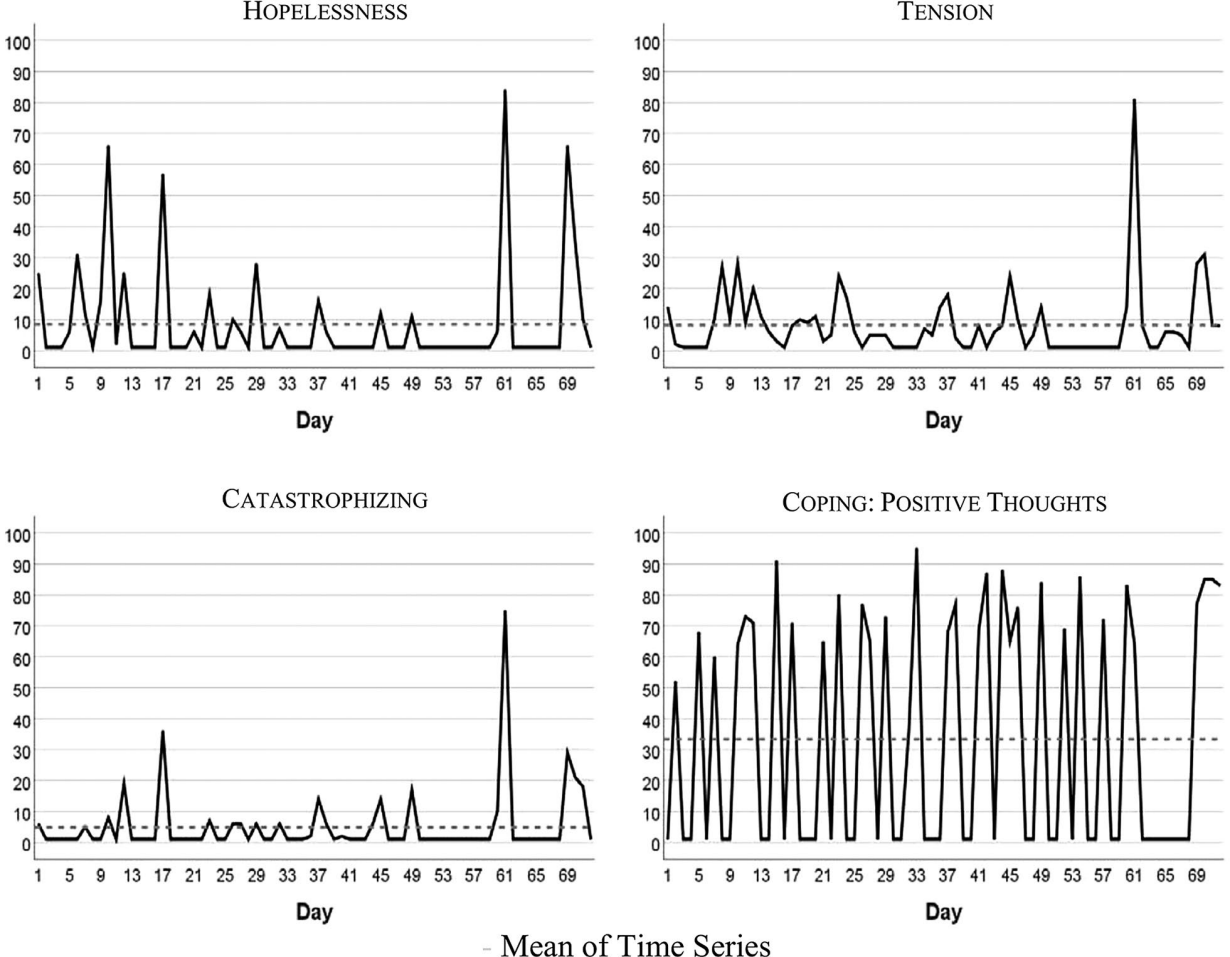
Time series of hopelessness, tension, catastrophizing, coping

Figure 1 visualizes the patient’s development of somatic symptoms, abdominal pain (AP), and daily impairment (DI) over 72 successive days together with their autocorrelation and cross-correlation function. In both series there appeared strong discomfort with values distinctly higher than 20 on 7 days. Almost 90% of the measurements varied between 1 and 20 on the 100-point scale. The average (Mean AP = 11.10, DI = 14.35) and variability (Standard Deviation: AP = 15.90, DI = 18.55) were higher for DI than AP (see also Table 2). Both time series exhibited no trends. Figure 2 shows the time series of additional psychological variables and coping strategies.

**Table 2:** Characteristics of somatic and psychological (including coping) time series used in the diary study

	Min	Max	Med	M	S	Lag AC	P ADF
**Somatic variables**							
Abdominal Pain (AP)	1	90	6.5	11.10	15.90	10	.03
IBS Associated Daily Impairment (DI)	1	87	8.5	14.35	18.55	1	.01
**Psychological variables**							
Nervousness (N)	1	86	10.5	14.40	14.75	1	.02
Tension (T)	1	81	5.0	8.24	11.59	–	.01
Depressiveness (D)	1	30	2.0	4.06	4.42	1	.03
Pain-associated discomfort (PD)	1	91	6.0	11.99	17.51	10	.02
**Coping strategies**							
Catastrophizing (C)	1	75	1.0	5.13	10.76	–	.01
Hopelessness (H)	1	84	1.0	8.46	16.71	–	.03
Coping: Positive thoughts (CPT)	1	95	1.0	33.32	37.25	7	.04
Coping: Imagining pain outside the body (CIP)	1	74	1.0	3.96	12.27	9	.01

Med, Median; M, Mean; S, Standard Deviation; Lag AC, lag number with significant autocorrelation; P ADF, p value of the Augmented Dickey–Fuller test with the alternative hypothesis “time series is stationary”. All the variables are quantified on a 1–101 numeric scale. For AP, DI, N, T, D, PD, C, and H a higher score reflects higher somatic or psychological burden. For CPT and CIP, a higher score reflects an increased use of coping strategies

The time series quantitatively reflect the free text descriptions of the patient. The highest scores in AP and DI were recorded between days 57 and 68 of the study period. In the free text passages of the diary the patient noted that she experienced the Christmas holidays (days 52–67) as a period of high stress and increased IBS pain. In addition, on days 59–61 she described the occurrence of menstrual cramps together with IBS-associated pain and impairment.

Table 2 summarizes characteristics of somatic and psychological and coping time series. In the majority of cases all of the series except “Coping with positive thoughts” (CPT) ranged between 1 and 20 on the 100-point scale, with high values observed about 10% of the time. CPT values alternated between very low and high with values equal on 1 out of 40 days, and values higher than 50 on 31 days. All series were stationary, i.e., exhibited no trends. Three series (tension, catastrophizing, and hopelessness) demonstrated no autocorrelations.

Table 3 shows instantaneous correlations between the somatic and psychological (including coping) time series. In most cases, strong and positive correlations were observed. Interestingly, the relationship between psychological and coping variables with DI was stronger than with AP. The amount of predicted variance (R^2^) from linear regressions with psychological and coping measures as dependent variables and somatic variables as predictors varied between 12 and 94%. The non-significant correlation between depressiveness and abdominal pain could be due to the very limited range of the variable depressiveness over the course of the 72 days.

**Table 3:** Significant instantaneous correlations of somatization with psychological and coping variables

	Abdominal Pain	Daily Impairment	R^2^
Nervousness (N)	.24	.63	.41
Tension (T)	.32	.66	.44
Depressiveness (D)	-	.47	.25
Pain-associated discomfort (PD)	.97	.61	.94
Catastrophizing (C)	.42	.77	.59
Hopelessness (H)	.53	.70	.52
Coping: Positive thoughts (CPT)	.43	.55	.34
Coping: Imagining pain outside the body (CIP)	-	.33	.12

R^2^, portion of predicted variance in psychological variables, from the regressions PV_t_ = β_1_AP_t_ + β_2_DI_t_ + e_t_ where PV, psychological/coping variable

Table 4 summarizes the significant results of the VAR analyses for interdependencies between abdominal pain and psychological distress or coping strategies; only statistically significant findings from calculations for all possible combinations of variables are provided. Identified lagged or temporal relations showed mostly the same direction, indicating that previous values in the somatic variable (AP) were predictive of values in the psychological variables or coping strategies. The variance decomposition estimates show that somatic symptoms in the psychological (and coping) time series explain 12% to 41% of variability.

**Table 4:** Significant temporal dependencies between psychological variables and abdominal pain

Psychological /coping variable	Type of dependency	VAR order	Granger- Causality Test		% FEVD h = 10	Instantaneous correlation
			F	p		
Nervousness (N)	SS → N	2	3.39	.04	.17	.24
Tension (T)	SS → T	2	9.49	< .01	.31	.32
Catastrophizing (C)	SS → C	2	16.3	< .01	.41	.42
Hopelessness (H)	SS → H	2	6.04	< .01	.35	.53
Coping: Positive Thoughts (CPT)	SS → CPT	1	8.09	< .01	.24	.43
	CPT → SS	1	4.34	.04	.06	
Coping: Imagining pain outside the body (CIP)	SS → CIP	2	3.74	.03	.12	.07

SS, somatic symptoms, measured by the item “How severe is your abdominal (tummy) pain”

A significant Granger Test implies that the first variable has impact on the second variable. The test statistic is F(df_1_,df_2_), where df_1_ is a number of tested restrictions (k) and df_2_ = 2 T − 4 k − 2 for bivariate VAR models, T is length of time series, k is order of VAR model. Forecast Error Variance (FEV) Decomposition estimates the amount of variance in a dependent variable, explained by a corresponding cause variable during a period h; h = 10 means 10 days

**Fig. 3:**
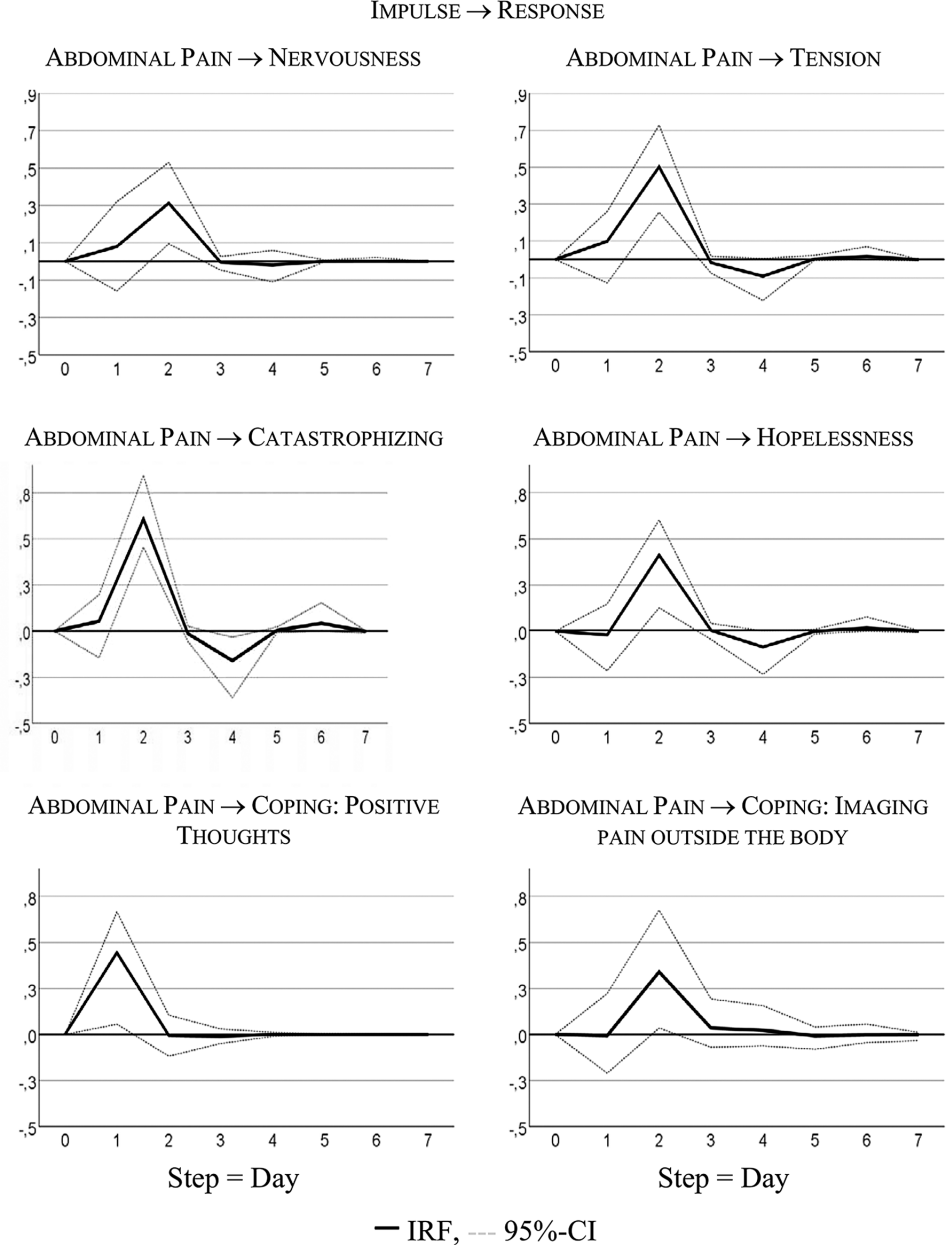
Time lagged psychological variables

Figure 3 visualizes responses of psychological states and coping strategies to increases in AP; it shows that psychological and coping aspects reacted with higher symptoms to an increase in AP. For instance, increasing AP caused a strong delayed increase in catastrophizing: + 0.60 standard deviations, i.e., about 7 points on the 100-point scale.

Table 4 shows that the bivariate system, including AP and CPT, is characterized by a bidirectional or feedback predictive causality. AP Granger-caused CPT with 24% of explained variance, CPT Granger-caused AP with 6% of explained variance. Both series also correlated instantaneously: r = 0.43, R² = 18%.

**Fig. 4:**
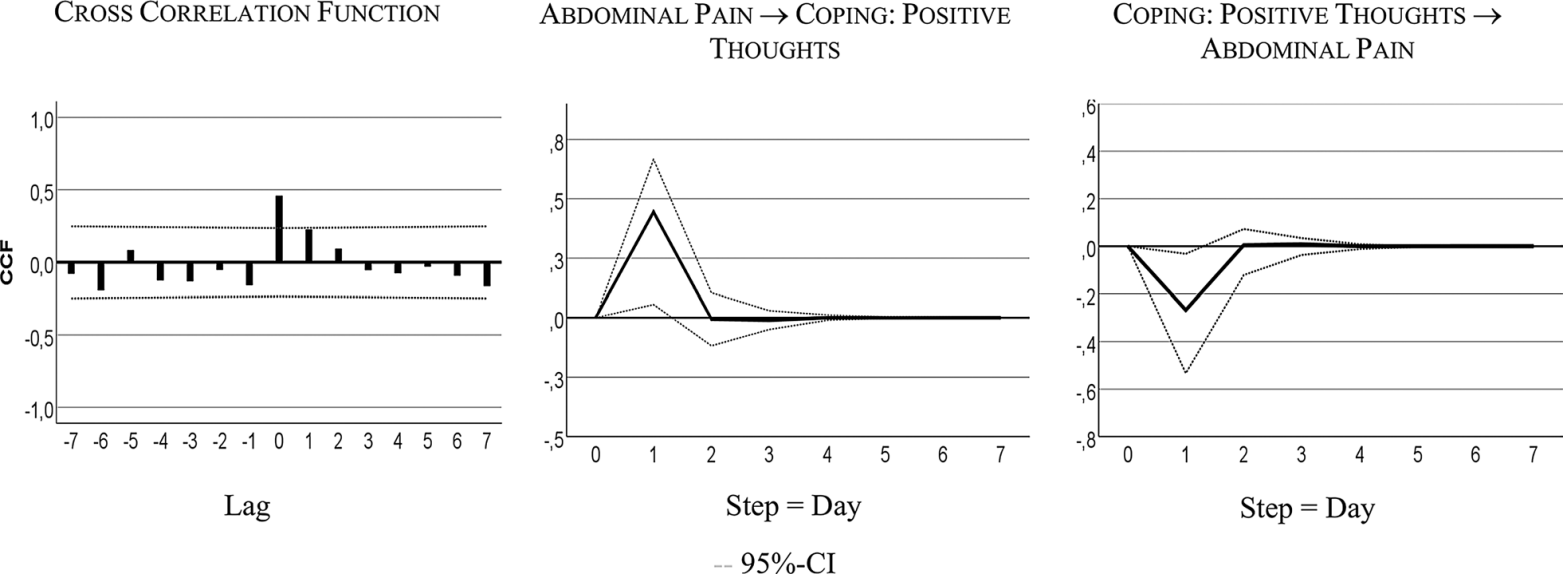
Cross-correlation and time lagged relationships: abdominal pain (AP) and coping with positive thoughts (CPT)

Figure 4 visualizes the feedback relationship. An increase in AP caused more CPT next day. Intensified CPT resulted in less pain on the subsequent day: i.e., a decrease of 0.25 standard deviations, 4-point on the 100-point scale.

## Discussion and conclusion

This is the first study to investigate the temporal relationships between somatic and psychological variables on a daily basis. We analyzed a female patient with IBS in her mid-twenties with symptoms of diarrhea, flatulence, and abdominal pain. She reported stress-related IBS symptoms as well as symptom related fears. In most variables, strong same-day correlations between somatic (especially daily impairment) and psychological (including coping) time series were observed. The day-lagged relationships indicated that higher values in abdominal pain on one day were predictive of higher values in psychological complaints (nervousness and tension) or of negative coping strategies (catastrophizing, hopelessness) on the following day. The use of positive thinking as a positive coping strategy was helpful in reducing the pain on the following days.

All variables remained stationary—that is, time series exhibited no trends over the measured time period (72 days). In the study period, the patient did not receive additional psychotherapeutic treatment, nor did she report long-lasting stressors. Therefore, we did not expect her symptoms to change over a longer period of time. The stability of IBS symptoms is supported by literature that usually describes IBS as a chronic disease. The diagnostic criteria for IBS also imply some symptom stability, because the symptoms must occur for a period of at least 3 months (with an onset at least 6 months prior the diagnosis) [22]. In addition, for IBS, population-based studies report a remission rate of about 55% only over a period of more than 10 years [26]. In addition to the general stationary trend of the variables, individual outliers with more severe symptoms were visible (e.g. the Christmas Holidays on days 52–67).

The patient stated that stressful or stress-free episodes would influence her symptoms; this was also reflected in the same-day analysis. In the free text of the diary the patient also described that in specific stressful situations she was ashamed of her symptoms and related consequences. The high same-day correlations between the somatic (AP, DI) and psychological time series (nervousness, tension, depressiveness, catastrophizing, hopelessness) reflect this interdependency—which the patient is aware of—between IBS symptoms and psychological state. Interestingly, this correlation was even higher for DI, meaning that functionality is especially important. The interaction between somatic and psychological distress is also described in previous studies. Midenfjord et al. (2019), for instance, showed in a cross-sectional study that IBS patients with psychological distress demonstrated more severe somatic symptoms and a lower quality of life [27]. Varni et al. (2017) found in a sample of pediatric patients with functional gastrointestinal disorders that somatic symptoms were differentially related to decreased health-related quality of life [28]. Another study reported a correlation between pain intensity and intensity of psychopathological symptoms (such as low spirits or anxiety) in IBS patients [29] while Dong et al. (2020) showed that IBS symptom severity predicted health-related quality of life influenced by stressful life events [30]. Interestingly, there is evidence that this association between current abdominal symptoms and psychological distress is not limited to functional gastrointestinal diseases but can also be seen in inflammatory bowel diseases [31]. The underlying physiological mechanism for the interaction between somatic and psychological distress could be explained by the concept of the (microbiome-) gut-brain axis. The (microbiome-)gut-brain axis refers to the complex network of connections between the microbiota, the enteric nervous system, and the central nervous system. [3, 4, 32, 33]. Previous research has shown that the link between gastrointestinal symptoms and psychological distress could be based on a complex and bidirectional interaction between biological, psychological, and social factors [5]. For example, visceral hypersensitivity and an enhanced perceptual response to gastrointestinal sensations can trigger gastrointestinal specific anxiety [5, 32]. On the other hand, psychosocial distress can lead, for instance, to an activation of the enteric and autonomic nervous system, which may trigger a change in smooth muscle activity or glandular secretion thus leading to IBS-symptoms. [32].

In addition to the daily correlation, it is also useful to look at day-to-day relationships in order to make time-delayed effects more visible and to answer the question whether or not psychological complaints precede IBS complaints, or vice versa. In literature, both perspectives are described for mental illnesses and IBS [6–8]. However, for this particular patient we found a strong time-delayed relationship between IBS symptoms, the following psychological complaints (nervousness, tension), and negative coping strategies (catastrophizing, hopelessness). This shows that having abdominal pain on one day was associated with more psychological stress the next day, not vice versa. This is in line with another study showing the temporal relationship that abdominal symptoms lead to increased stress and negative affect, while increased daily life stressors even lowered the IBS-symptoms [34]. This is interesting, as in literature frequently the opposite temporal direction or a feedback-loop is assumed [35]. Patel et al. (2016), for instance, investigated the relationship between sleep, mood and somatic symptoms in a sample of IBS patients and healthy controls over the course of 7 days [36]. In IBS patients, sleep disturbances were predictive for abdominal pain on the following day. Additional analyses showed that the sleep effects on abdominal pain in IBS patients could be mediated by depression and anxiety [36].

The question arises why our data show that the patient first develops gastrointestinal complaints and only afterwards psychological complaints. The patient herself had the impression that increased stress would lead to an increase in symptoms. For instance, during the short stressful event of applying for a new job the patient reported an onset of IBS complaints. She also reported that in this case the immediate application of a coping strategy (such as calming down) had helped her to reduce the symptoms. However, this sequence occurred over the course of only several hours—and would thus be reflected in the high same-day correlations of the time series (and not in the day-lagged correlations). On the other hand, shorter time intervals had been tested in Chan's study with an outcome similar to ours [34]. It is also possible that shorter daily stressors could also lead to a distraction from the IBS-symptoms, while longer stressors (like Christmas Holidays in the case of our study) may lead to an increase in symptoms.

Another interesting approach to feelings and symptoms of IBS is the concept of alexithymia. This concept states, among others, that feelings in IBS-patients may be misinterpreted as negative bodily sensations [37]. For our patient, this could mean that in stressful situations (such as job search or exam phases) she may initially perceive her feelings only physically and interpret them as a preliminary stage of a new outbreak of her IBS. The hyper-focus on the symptoms could initially intensify them. Shortly afterwards, the patient may get negative feelings from the IBS symptoms themselves.

The time-lagged correlation between IBS complaints and the following psychological complaints and negative coping strategies could be related to the patient’s social anxiety and the pressure to perform. In the free text of the diary the patient described that with the occurrence of abdominal complaints she would fear that soft bowel movements would follow, and that she would not be able to reach a toilet in a timely manner; she also felt ashamed when she had to leave certain events because of her IBS symptoms. Physiologically, this relationship between IBS complaints and following psychological distress could again be explained by the (microbiome-)gut-brain axis [5, 32]. The occurrence of abdominal complaints (maybe as an expression of visceral hypersensitivity) can trigger gastrointestinal specific anxiety and the autonomic nervous systems as well as the hypothalamic pituitary axis are sending stress signals to the gut, resulting, among others, in a higher bowel motility and secretion leading to diarrhea and pain [32].

Interestingly, abdominal pain was not associated with a depressive feeling in general, but with negative processing (such as hopelessness and catastrophizing) as well as tense or anxious arousal (nervousness, tension). These negative feelings and coping strategies had no effect on the patient’s increased abdominal pain the next day; in contrast, the use of positive coping strategies was helpful.

The patient reported using positive coping strategies to reduce her symptoms; this was also seen in the data analysis. The intensified use of a specific coping strategy on one day (thinking of things the patient enjoyed doing) was followed by a decrease in pain on the subsequent day. Conversely, an increase in pain was followed by an increased use of this coping strategy. This corresponds to the clinical impression and the self-report of the patient: She considered relaxation techniques and new coping strategies such as distraction as beneficial for her condition. This result is supported by literature that considers psychotherapeutic treatment, including positive coping strategies, as a possible treatment of IBS [38].

In summary, the results of the time series analysis partly reflect the self-report of the patient as well as the clinical impression of the outpatient caretaker. However, our results expand upon these insights by showing temporal relationships between IBS symptoms and psychological variables over consecutive days—with psychological changes following changes in abdominal pain and related impairment. In addition, a mutual day-lagged relationship between IBS symptoms and coping could be detected.

This study has several implications: Overall, it shows that at the very least this patient is aware of her individual process of personal change, her fears, and her coping strategies––all of which to a large extent, could be confirmed by the time series analysis––an analysis that also provided additional information. This supports the hypothesis that individual characterizations are promising in terms of providing a better understanding of specific mechanisms, as well as an understanding of how temporal interactions between IBS symptoms and psychological symptoms are related. In clinical practice, practitioners should consider individual explanatory models of aggravating factors and coping strategies and stay open to psychosomatic as well as somatopsychic mechanisms. Previous psychological treatment recommendations for IBS patients concluded that a change in illness-specific cognitions as well as gastrointestinal anxiety as key mechanisms may have an effect on the outcomes of IBS symptom severity and quality of life [39]. In this case study, only positive thinking had a time-lagged effect on a decrease in abdominal pain, while catastrophizing and hopelessness were a result of having abdominal pain previously. Although it is not possible to generalize the results of an individual case, this supports the fact that treatments which more directly target abdominal symptoms (e.g., hypnotherapy) may have promising effects on IBS symptoms as well as associated psychological complaints. Therefore, a disorder-oriented integrative group intervention for IBS with gut-directed hypnotherapy seems promising [15].

From a methodological point of view, we have to point out that the here applied concept of Granger-causality does not equal causality. Causality according to Hill [40] can be assessed by using the following 9 criteria: strength, consistency, specificity, temporality, biological gradient, plausibility, coherence, experiment, analogy. The definition of Granger-causality, however, implies only that previous values of a time series X (e.g. somatic symptoms) improve prediction of future values of another series Y (e.g. nervousness of the patient). It does not imply the causality of X for Y.

Our study has several limitations. Firstly, we examined only one patient suffering from IBS; the generalizability of the results is therefore limited. We cannot simply transfer the results to other IBS patients but must carefully investigate further patient samples in regard to temporal relationships and interactions between somatic and psychological variables. Secondly, we were able to detect day-to-day changes only; shorter periods of time could not be captured. Nevertheless, previous studies mainly focused on longer time periods which is why this approach is still more advantageous in terms of capturing the direct relationships. Nevertheless, we were able to show a clear picture of a single IBS-patient. This is helpful as IBS is a complex illness with, in all likelihood, heterogeneous genesis and factors. A comprehensive case study could help identify subclasses of IBS to arrive at a better treatment and avoid dilution effects.

In conclusion we found in the presented case that somatic symptoms temporally precede psychological complaints. In addition, for this patient, the use of positive thoughts as a coping strategy was helpful in reducing pain. Further analyses should be conducted to verify if these relationships can be found in other patients who suffer from IBS symptoms.

## Data Availability

The datasets used and analysed during the current study are available from the corresponding author on reasonable request.

## Abbreviations

IBS: Irritable bowel syndrome
AP: Abdominal pain
DI: IBS-associated daily impairment
N: Nervousness
T: Tension
D: Depressiveness
PD: Pain-associated discomfort
C: Catastrophizing
H: Hopelessness
CPT: Coping with positive thoughts
CIP: Coping with imagining pain outside the body
